# Anemia risk factors among people living with HIV across the United States in the current treatment era: a clinical cohort study

**DOI:** 10.1186/s12879-020-04958-z

**Published:** 2020-03-20

**Authors:** B. N. Harding, B. M. Whitney, R. M. Nance, S. A. Ruderman, H. M. Crane, G. Burkholder, R. D. Moore, W. C. Mathews, J. J. Eron, P. W. Hunt, P. Volberding, B. Rodriguez, K. H. Mayer, M. S. Saag, M. M. Kitahata, S. R. Heckbert, J. A. C. Delaney

**Affiliations:** 1 Department of Medicine, University of Washington, 1959 NE Pacific Street, Health Sciences Building F-26, Box 357236, Seattle, WA 98195 USA; 2 University of Alabama Birmingham, Birmingham, USA; 3 Johns Hopkins University, Baltimore, USA; 4 University of California San Diego, San Diego, USA; 5 University of North Carolina, Chapel Hill, USA; 6 University of California San Francisco, San Francisco, USA; 7 Case Western Reserve University, Cleveland, USA; 8 Fenway Health Institute, Boston, USA

**Keywords:** Anemia, Hemoglobin, Cohort, HIV

## Abstract

**Background:**

Anemia is common among people living with HIV infection (PLWH) and is associated with adverse health outcomes. Information on risk factors for anemia incidence in the current antiretroviral therapy (ART) era is lacking.

**Methods:**

Within a prospective clinical cohort of adult PLWH receiving care at eight sites across the United States between 1/2010–3/2018, Cox proportional hazards regression analyses were conducted among a) PLWH free of anemia at baseline and b) PLWH free of severe anemia at baseline to determine associations between time-updated patient characteristics and development of anemia (hemoglobin < 10 g/dL), or severe anemia (hemoglobin < 7.5 g/dL). Linear mixed effects models were used to examine relationships between patient characteristics and hemoglobin levels during follow-up. Hemoglobin levels were ascertained using laboratory data from routine clinical care. Potential risk factors included: age, sex, race/ethnicity, body mass index, smoking status, hazardous alcohol use, illicit drug use, hepatitis C virus (HCV) coinfection, estimated glomerular filtration rate (eGFR), CD4 cell count, viral load, ART use and time in care at CNICS site.

**Results:**

This retrospective cohort study included 15,126 PLWH. During a median follow-up of 6.6 (interquartile range [IQR] 4.3–7.6) years, 1086 participants developed anemia and 465 participants developed severe anemia. Factors that were associated with incident anemia included: older age, female sex, black race, HCV coinfection, lower CD4 cell counts, VL ≥400 copies/ml and lower eGFR.

**Conclusion:**

Because anemia is a treatable condition associated with increased morbidity and mortality among PLWH, hemoglobin levels should be monitored routinely, especially among PLWH who have one or more risk factors for anemia.

## Background

Anemia (hemoglobin < 10 g/dL) and severe anemia (hemoglobin< 7.5 g/dL) [1] are common among people living with HIV (PLWH) [2], and the prevalence of anemia increases with HIV disease severity [3, 4]. While estimates of anemia prevalence vary depending on factors including age, setting, HIV disease stage, use of antiretroviral therapy (ART), sex, and injection drug use status [2, 3], it is estimated that 18–32% of PLWH without AIDS and 48–85% of PLWH with clinical AIDS have anemia in the United States, compared to 15–17% of people living without HIV [3, 5]. Many of the aforementioned prevalence estimates date from before or early in the ART era before the current treatment initiation guidelines and improvements in HIV care [6].

Anemia is thought to be an independent prognostic indicator among PLWH, associated with HIV disease progression [2, 7]. Furthermore, previous studies have reported associations between anemia and mortality [4, 8–10], health-related quality-of-life, dementia [11], and treatment failure [12] among PLWH.

The causes of anemia among PLWH are multifactorial. Immune dysregulation during HIV infection can increase anemia risk through red blood cell destruction (hemolysis) or ineffective red blood cell production, which are influenced by infections of the spleen or circulatory system [13]. Blood loss among PLWH is not uncommon and may occur as a result of neoplastic disease or gastrointestinal lesions accompanying opportunistic infections. Additional mechanisms for HIV-related anemia include deficiencies of vitamin B12, folate, or iron [13].

The goal of this study was to estimate the association between patient characteristics and (a) risk of anemia, (b) risk of severe anemia, or (c) a lower hemoglobin level to identify risk factors for anemia among a large cohort of PLWH within the US in the modern HIV treatment era.

## Methods

### Overview and setting

**Fig. 1:**
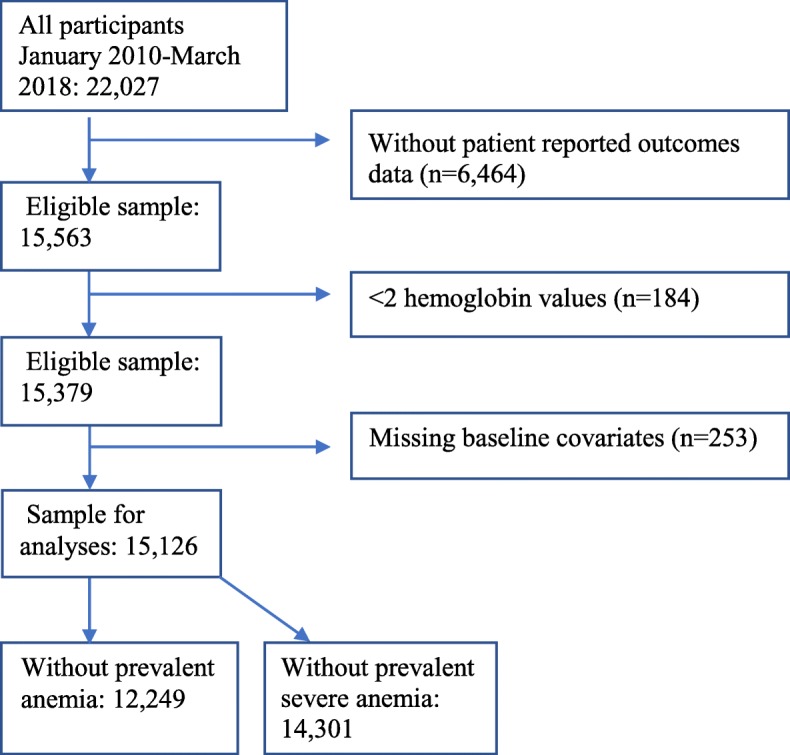
Flow chart for inclusion/exclusion criteria

This study included PLWH in care in the Centers for AIDS Research (CFAR) Network of Integrated Clinical Systems (CNICS) cohort during January 1, 2010 to March 31, 2018. The CNICS cohort has been described in detail elsewhere [14]. Briefly, CNICS is a dynamic prospective clinical cohort of more than 32,000 PLWH ≥18 years of age receiving care at eight participating academic sites across the US. Comprehensive clinical data, including diagnoses, medications (including ART), laboratory test results, demographic information, and historical information, including ART use before enrollment, are collected through electronic medical records and other institutional data systems at each site and harmonized in the CNICS data repository. Medication data, including ART use, is entered into the electronic medical records by clinicians or prescription fill/refill data are uploaded directly from Pharmacy Systems and verified through medical record review. Patient reported measures and outcomes data (PROs) are collected in the CNICS assessment as part of routine clinical care appointments [15, 16]. However, sites varied in their year of PRO initiation, so it is available for the majority but not all CNICS participants. The CNICS clinical assessment of PROs includes measures of smoking, alcohol use and illicit drug use. Participants entered the present study on January 1, 2010 or the earliest date that they had been enrolled in CNICS for 6 months, whichever came later (baseline). Additional exclusions were made for participants without PRO data, or for those with less than two available hemoglobin lab values during the study years (Fig. 1). Informed consent was obtained from all participants to be part of CNICS and institutional review boards at each site approved CNICS protocols.

### Participant characteristics

Characteristics that were analyzed as potential risk factors for anemia, severe anemia or a lower hemoglobin level included the following characteristics analyzed at baseline: age, sex, race/ethnicity, BMI (closest available BMI, categorized as < 18.5, 18.5- < 25, 25- < 30, ≥30 kg/m^2^), smoking status (never, former, current), hazardous alcohol use (a score of ≥4 for women or ≥ 5 for men on the Alcohol Use Disorders Identification Test (AUDIT-C) scale [17]) illicit drug use (use of cocaine/crack, methamphetamines/crystal and/or illicit opioids, using the Alcohol, Smoking and Substance Involvement Screening Test (ASSIST) [18]). In addition, the following characteristics were analyzed as potential time-updated risk factors: hepatitis C virus (HCV) coinfection, kidney function measured using estimated glomerular filtration rate (eGFR, categorized as < 30, 30–59, or ≥ 60 mL/minute/1.73 m^2^), CD4 cell count (categorized as ≥500, 350–499, 200–399, 100–199 or < 100 cells/mm^3^), detectable viral load (VL, ≥400 copies/ml), treatment with ART and time in care at CNICS site, defined as time from enrollment in CNICS until the last available CNICS activity (either last lab date or last visit). In addition, we adjusted for CNICS site. All covariates were selected a priori, based on review of the literature and clinical knowledge.

### Outcome ascertainment

Hemoglobin levels, expressed in grams per deciliter (g/dL), were ascertained using inpatient and outpatient laboratory data obtained as part of routine clinical care. Outcomes included incident anemia (the first hemoglobin measure below 10 g/dL), incident severe anemia (the first hemoglobin measure below 7.5 g/dL), and changes in hemoglobin level. Additional interest lay in investigating associations between patient characteristics and chronic anemia, defined as anemia lasting for at least 6 months, identified using hemoglobin lab results on two separate occasions at least 6 months apart which were consistently in the anemic range without any additional hemoglobin values above the anemia range during that six-month period.

### Statistical analysis

Two multivariable Cox proportional hazards regression analyses were conducted. First, among the subset of PLWH who did not have a history of anemia at baseline, associations were measured between risk factors and development of anemia. Second, among the subset of participants who did not have a history of severe anemia at baseline, associations were measured between risk factors and development of severe anemia. Participants were censored at (a) the time they developed anemia (for the incident anemia analyses) or at the time they developed severe anemia (for incident severe anemia analyses), (b) at the time of last activity in CNICS, (c) at the time of death or (d) at the time of administrative censoring due to end of follow-up per site, whichever came first. The timescale for the models was time since baseline. In post hoc analyses, we estimated the risk of anemia for subgroups identified as having combinations of high risk characteristics and tested for interaction on a multiplicative scale.

Linear mixed effects models were used to examine the associations between patient characteristics and hemoglobin levels among all PLWH. Mixed-effects models utilize random intercepts and slopes for time at the participant level to handle irregular patterns of repeated measures over follow-up [19].

### Sensitivity analysis

In a sensitivity analysis, we assessed associations between patient characteristics and chronic anemia (defined as anemia lasting for at least 6 months) using a Cox proportional hazards regression model among the population who were anemia-free at baseline. Because factors such as severe bleeding, blood donation and blood transfusion could impact hemoglobin levels at a given time point and are difficult to capture, this sensitivity analysis allowed us to describe associations among those with chronic anemia.

All analyses were performed using Stata version 14.2.

## Results

There were 15,126 PLWH who were included in these analyses (Fig. 1). A total of 12,249 (81%) did not have a history of anemia at baseline, and 14,301 (95%) did not have a history of severe anemia at baseline. Participants had a median of 14 hemoglobin values measured during a median follow-up of 6.6 (interquartile range [IQR] 4.3–7.6) years. Table 1 provides baseline characteristics for all study participants, as well as characteristics for the 1086 participants who developed anemia and 465 participants who developed severe anemia during follow-up. For the overall population, the median age of study participants was 44 (IQR 35–51) years at baseline, 17% were female, 17% were co-infected with HCV, and 80% were using ART.

**Table 1:** Baseline demographic and clinical characteristics of PLWH in care at sites across the United States overall and by anemia status^a^

**Participant characteristics**	**All participants (*N*** **= 15,126)**	**Developed anemia during follow-up (*n*** **= 1086)**	**Developed severe anemia during follow-up (*n*** **= 465)**
Age (median, IQR)	44 (35–51)	46 (39–53)	46 (39–53)
Female	2620 (17)	303 (28)	156 (34)
Race/ethnicity			
White	6712 (44)	411 (38)	148 (32)
Black	5560 (37)	529 (49)	243 (52)
Hispanic	2145 (14)	108 (10)	56 (12)
Other/missing	709 (5)	38 (3)	18 (4)
Years in CNICS at baseline (median, IQR)	1.1 (0.5–6.0)	2.2 (0.5–7.0)	3.0 (0.5–7.7)
Viral load ≥400 copies/ml	3314 (22)	338 (31)	170 (37)
CD4 count (cells/mm^3^)			
< 100	777 (5)	107 (10)	78 (17)
100–199	1236 (8)	99 (9)	64 (14)
200–349	2686 (18)	219 (20)	122 (26)
350–499	3323 (22)	233 (22)	79 (17)
≥ 500	7104 (47)	428 (39)	122 (26)
Hepatitis C virus coinfection	2556 (17)	321 (30)	146 (31)
Kidney function (eGFR)			
< 30	164 (1)	25 (2)	23 (5)
30–59	620 (4)	51 (5)	34 (7)
≥ 60	14,342 (95)	1010 (93)	408 (88)
BMI (kg/m^2^)			
< 18.5	352 (2)	24 (2)	20 (4)
18.5 to < 25.0	6494 (43)	472 (44)	207 (44)
25.0 to < 30.0	5199 (34)	339 (31)	128 (28)
≥ 30.0	3081 (21)	251 (23)	110 (24)
ART use	12,070 (80)	821 (76)	351 (75)
MCV (fL)	91 (87–95)	91 (86–96)	90 (85–96)
Smoking status			
Never	6211 (41)	408 (38)	188 (41)
Former	3492 (23)	195 (18)	80 (17)
Current	5423 (36)	483 (44)	197 (42)
Hazardous alcohol use^b^	4239 (28)	244 (22)	110 (24)
Illicit drug use^c^	2283 (15)	165 (15)	64 (14)

^a^ Baseline was defined as the earliest date during January 1, 2010- March 31, 2018 that a person had 6+ months in CNICS care

^b^ Hazardous alcohol use defined a score of ≥4 for women or ≥ 5 for men on the AUDIT-C scale

^c^ Use of cocaine/crack, methamphetamines/crystal, and/or illicit opioids

Abbreviations: *ART* antiretroviral therapy, *BMI* body mass index, *eGFR* estimated glomerular filtration rate, *MCV* mean corpuscular volume *PLWH* people living with HIV

The incidence of anemia was 1.95/100 person-years and the incidence of severe anemia was 0.68/100 person-years. Characteristics associated with incident anemia included older age, female sex, black race/ethnicity, HCV coinfection, lower CD4 cell count, higher viral load, and lower eGFR, indicative of poor kidney function (Table 2). Risk factors associated with severe anemia were similar to those for anemia with the exception of older age and black race/ethnicity (Table 2). In post hoc analyses, we estimated the risk of anemia for subgroups identified as having combinations of high risk characteristics, including CD4 < 100, female sex, and coinfection with HCV. Among women, CD4 < 100 as compared with CD4 ≥ 500 was associated with a HR for incident anemia of 10.64 (95% CI 8.41, 13.47), while for men the HR was 4.11 (2.60, 6.52); *p*-value for low CD4-sex interaction < 0.001; Supplemental Table 1). Among those with HCV coinfection, CD4 < 100 was associated with a HR for incident anemia of 10.20 (95% CI 8.01, 12.98), while for those without HCV coinfection, the HR was 5.01 (95% CI 3.39, 7.42; p-value for low CD4-HCVcoinfection interaction =0.001; Supplemental Table 2).

**Table 2:** Factors associated with developing anemia or severe anemia in multivariable adjusted analyses among those free of anemia (*n* = 12,249) or among those free of severe anemia (*n* = 14,301) at baseline

**Participant characteristics**	**Anemia** ***n*** **= 12,249** **Hazard ratio (95%CI)**	**Severe anemia** ***n*** **= 14,301** **Hazard ratio (95% CI)**
Age per 10 years	1.11 (1.04, 1.18)	1.05 (0.95, 1.16)
Female (ref male)	2.45 (2.12, 2.82)	2.09 (1.69, 2.58)
Race/ethnicity (ref white)		
Black	1.19 (1.03, 1.39)	0.83 (0.65, 1.05)
Hispanic	0.90 (0.72, 1.13)	1.06 (0.76, 1.47)
Other/missing	0.99 (0.71, 1.39)	1.13 (0.69, 1.86)
Hepatitis C virus coinfection	1.79 (1.56, 2.05)	1.62 (1.31, 1.99)
CD4 cell count (cells/mm3) (ref ≥500)		
350–499	1.38 (1.16, 1.65)	2.23 (1.68, 2.97)
200–349	1.67 (1.39, 2.02)	2.39 (1.78, 3.22)
100–199	2.83 (2.28, 3.62)	3.89 (2.77, 5.45)
< 100	8.40 (6.85, 10.51)	15.53 (11.56, 20.92)
Viral load ≥400 copies/ml	1.43 (1.23, 1.64)	1.86 (1.48, 2.34)
Kidney function (eGFR) (ref > 60)		
30–60	2.35 (1.91, 2.89)	2.67 (1.97, 3.63)
< 30	17.84 (13.94, 24.02)	23.01 (17.85, 29.68)
ART use	0.92 (0.80, 1.05)	1.00 (0.81, 1.23)
BMI (ref 18.5- < 25)		
< 18.5	0.89 (0.59, 1.32)	1.23 (0.77, 1.96)
25.0 to < 30.0	0.88 (0.77, 1.02)	0.73 (0.58, 0.91)
≥ 30.0	1.02 (0.87, 1.20)	0.92 (0.72, 1.17)
Smoking status (ref never)		
Former	0.89 (0.75, 1.06)	0.82 (0.63, 1.07)
Current	1.08 (0.94, 1.25)	0.90 (0.72, 1.12)
Hazardous alcohol use^a^	0.93 (0.81, 1.06)	1.07 (0.87, 1.31)
Illicit drug use^b^	0.99 (0.86, 1.14)	0.99 (0.77, 1.21)

^a^ Hazardous alcohol use defined a score of ≥4 for women or ≥ 5 for men on the AUDIT-C scale

^b^ Use of cocaine/crack, methamphetamines/crystal, and/or illicit opioids

Abbreviations: ART: antiretroviral therapy; BMI: body mass index; eGFR: estimated glomerular filtration rate

Among the 12,249 PLWH who were free of anemia at baseline, 265 developed chronic anemia (lasting for at least 6 months), during follow-up for an incidence of 0.46/100 person-years. For chronic anemia, associations with patient characteristics were similar to those in the primary analysis of anemia. (Table 3).

**Table 3:** Factors associated with developing chronic anemia in analyses among those without anemia at baseline; *n* = 12,249

**Participant characteristics**	**Hazard ratio**	**95% CI**
Age per 10 years	1.18	1.04, 1.34
Female (ref male)	3.25	2.48, 4.27
Race/ethnicity (ref white)		
Black	1.96	1.43, 2.68
Hispanic	1.29	0.82, 2.04
Other/missing	1.12	0.54, 2.33
Hepatitis C virus coinfection	1.76	1.32, 2.36
CD4 cell count (cells/mm3) (ref ≥500)		
350–499	1.10	0.77, 1.56
200–349	1.93	1.40, 2.67
100–199	1.83	1.17, 2.88
< 100	4.11	2.67, 6.33
Viral load ≥400 copies/ml	1.86	1.48, 2.34
Kidney function (eGFR) (ref > 60)		
30–60	1.31	0.72, 2.37
< 30	5.40	2.35, 12.42
ART use	0.99	0.73, 1.35
BMI (ref 18.5- < 25)		
< 18.5	0.75	0.31, 1.85
25.0 to < 30.0	0.85	0.64, 1.14
≥ 30.0	0.82	0.59, 1.15
Self-reported smoking (ref never)		
Former	0.82	0.57, 1.16
Current	0.93	0.70, 1.24
Hazardous alcohol use^a^	0.86	0.66, 1.12
Illicit drug use^b^	0.87	0.64, 1.18

^a^ Hazardous alcohol use defined a score of ≥4 for women or ≥ 5 for men on the AUDIT-C scale

^b^ Use of cocaine/crack, methamphetamines/crystal, and/or illicit opioids

Abbreviations: *ART* antiretroviral therapy, *BMI* body mass index, *eGFR* estimated glomerular filtration rate

Average hemoglobin levels remained steady during follow-up; the median level was 14.3 g/dL (IQR 13.1–15.2) at baseline and 14.3 g/dL (IQR 13.1–15.3) at the last available measurement per person. Characteristics that were associated with having a lower hemoglobin level measured during follow-up time included: older age, female sex, non-white race/ethnicity, HCV coinfection, lower CD4 cell count, higher VL, lower eGFR, ART use, illicit drug use and BMI < 18.5; conversely, BMI between 25 to < 30 or BMI ≥ 30 was associated with higher hemoglobin overtime. Other factors associated with a higher hemoglobin level included being a current smoker and reporting hazardous alcohol use (Table 4).

**Table 4:** Characteristics associated with hemoglobin level over follow-up time in analyses (linear mixed-effect model); *N* = 15,126

**Participant characteristics**	**Coeff**^**a**^	**95% CI**	***P*-value**
Age per 10 years	− 0.01	− 0.01, − 0.01	< 0.001
Female (ref male)	−1.68	− 1.74, − 1.62	< 0.001
Race/ethnicity (ref white)			
Black	−0.75	− 0.80, − 0.70	< 0.001
Hispanic	− 0.15	− 0.21, − 0.08	< 0.001
Other/missing	−0.13	− 0.23, − 0.03	0.011
Hepatitis C virus coinfection	− 0.21	−0.25, − 0.16	< 0.001
CD4 cell count (cells/mm3) (ref ≥500)			
350–499	−0.18	− 0.19, − 0.16	< 0.001
200–349	−0.39	− 0.41, − 0.37	< 0.001
100–199	−0.81	− 0.83, − 0.78	< 0.001
< 100	−1.49	− 1.52, − 1.46	< 0.001
Viral load ≥400 copies/ml	−0.09	− 0.11, − 0.07	< 0.001
Kidney function (eGFR) (ref > 60)			
30–60	− 0.15	−0.16, − 0.13	< 0.001
< 30	−1.11	− 1.15, − 1.07	< 0.001
ART use	− 0.03	−0.04, − 0.01	< 0.001
BMI (ref 18.5- < 25)			
< 18.5	−0.24	− 0.37, − 0.10	0.001
25.0 to < 30.0	0.24	0.19, 0.29	< 0.001
≥ 30.0	0.16	0.10, 0.22	< 0.001
Self-reported smoking (ref never)			
Former	0.05	−0.01, 0.11	0.055
Current	0.15	0.10, 0.20	< 0.001
Self-reported hazardous alcohol use^b^	0.13	0.09, 0.18	< 0.001
Self-reported illicit drug use^c^	−0.19	−0.24, − 0.14	< 0.001

^a^Coefficient is the mean difference per year in hemoglobin (g/dL) for each variable after adjustment for all other covariates presented in the table

^b^ Hazardous alcohol use defined a score of ≥4 for women or ≥ 5 for men on the AUDIT-C scale

^c^ Use of cocaine/crack, methamphetamines/crystal, and/or illicit opioids

Abbreviations: *ART* antiretroviral therapy, *BMI* body mass index, *eGFR* estimated glomerular filtration rate

## Discussion

Anemia is an important clinical condition among PLWH. In this study of 15,126 PLWH in care in the current treatment era (2010 and after) in the US, we observed that factors including older age, female sex, black race, HCV coinfection, lower CD4 cell counts, VL ≥400 copies/ml and lower eGFR were associated with an increased risk of anemia.

Lower CD4 cell count has been previously identified as a risk factor for anemia. The present study confirmed these findings, however we report much larger increased risks of anemia (HR = 8.40, 95%CI: 6.85, 10.51) and severe anemia (HR = 15.53, 95%CI:11.56–20.92) among PLWH with CD4 cell counts < 100 cells/mm^3^ than have been previously reported [20–24]. Risks this large in magnitude suggest that additional screening and careful monitoring would potentially be useful among participants with CD4 levels < 100 cells/mm^3^. In addition, we found that those with CD4 levels < 100 who were female or who also had HCV coinfection were at an especially high risk of anemia.

Similar to the present study, previous work has found that comorbidities such as HCV infection [25, 26] and poor kidney function [27] are associated with anemia risk. Furthermore, the current recommended treatment for chronic HCV has known side effects of reduced hemoglobin levels and reversible anemia [25, 26]. Additionally, high BMI has been associated with iron [28] and vitamin B12 deficiencies [29] and a lower chance of recovery from anemia among PLWH [30]. However, we did not find strong evidence in the present study that BMI is associated with anemia risk.

Prior work has also shown that smoking leads to increased hemoglobin levels and a reduction in the detection of anemia because smoking reduces the oxygen carrying capacity of red blood cells, to which the body responds to by increasing hemoglobin levels as a means to counteract this reduction in oxygen carrying capacity [31]. However, limited research exists into potential associations between the use of other substances including alcohol or illicit drugs and anemia. We found that being a current smoker was associated with small increases in hemoglobin over time, but did not detect any measurable differences in anemia risk related to current smoking, alcohol or other substance use in the present study.

In the early treatment era, use of zidovudine (AZT), was a known cause of bone marrow suppression [32]. In more recent years, AZT use has decreased substantially as other, better-tolerated ART medications have become available. Though prior research has found that being on ART is associated with lower anemia incidence and severity for most PLWH [23, 33–35] due to the ability of ART to control HIV disease progression, we did not find an association between ART use and a reduction in anemia or severe anemia incidence in the present study. This may be explained by possible pro-anemic side effects of certain types of currently used ART regimens [36].

Strengths of this study include the large and geographically diverse study population, longitudinal data structure, and availability of information on behaviors including smoking, alcohol use, and illicit drug use. However, there are important limitations of this study, including our inability to classify anemia based on red cell indices, lack of information on hemoglobinopathies, nutritional deficiencies, chemotherapy or other medications with bone marrow toxicity, instances of severe bleeding, blood donation or blood transfusion, all of which could impact hemoglobin levels at a given time point, and the possibility of residual confounding due to the observational nature of the study. Additionally, we relied on self-report of smoking and substance use, which may be underreported. Finally, because this study was conducted among PLWH in care in the United States, our findings may not generalize to PLWH who are not in care or reside outside of the United States.

## Conclusions

In conclusion, we found that factors including older age, female sex, black race, HCV coinfection, lower CD4 cell counts, VL ≥400 copies/ml, and lower eGFR were associated with an increased risk of anemia. Because anemia is a treatable condition associated with increased morbidity and mortality among PLWH, clinicians should be aware of these risk factors. Hemoglobin levels should be monitored routinely, especially among PLWH who have one or more of the risk factors for anemia so that treatment can be initiated if deemed necessary.

## Supplementary information

**Additional file 1:Table S1.** Stratified table of anemia risk for CD4 < 100 and sex.

**Additional file 2:Table S2.** Stratified table for anemia risk and CD4 < 100 and HCV coinfection.

## Data Availability

The data that support the findings of this study are available from the CNICS Research Coordinating Committee, but restrictions apply to the availability of these data, which were used under license for the current study, and so are not publicly available. Data are however available from the authors upon reasonable request and with permission of CNICS Research Coordinating Committee.

## Abbreviations

PLWH: People living with HIV
ART: Antiretroviral therapy
CFAR: Centers for AIDS Research
CNICS: CFAR Network of Integrated Clinical Systems
PRO: Patient reported measures and outcomes data
BMI: Body mass index
AUDIT-C: Alcohol Use Disorders Identification Test
ASSIST: Alcohol, Smoking and Substance Involvement Screening Test
HCV: Hepatitis C virus
eGFR: estimated glomerular filtration rate
VL: Viral load
IQR: Interquartile range
AZT: zidovudine
