# Supplementary material for: Anemia risk factors among people living with HIV across the United States in the current treatment era: a clinical cohort study
Source: BMC Infect Dis. 2020 Mar 20;20:238. doi: 10.1186/s12879-020-04958-z (PMC7085166; doi:10.1186/s12879-020-04958-z)
Supplement: Supplementary file 1 — Additional file 1:Table S1. Stratified table of anemia risk for CD4 < 100 and sex. [file 12879_2020_4958_MOESM1_ESM.docx]

Supplemental Table 1: Stratified table of anemia risk for CD4<100 and sex

|  | Hazard Ratio (95%CI) | |
| --- | --- | --- |
|  | Female (n=1,568, n with CD4<100 =126) | Male (n=10,681, n with CD4<100 =948) |
| CD4<100 (ref CD4≥500) | 10.64 (8.41, 13.47) | 4.11 (2.60, 6.52) |
| Interaction between CD4<100 and sex, p=<0.001 | | |
