# Supplementary material for: Anemia risk factors among people living with HIV across the United States in the current treatment era: a clinical cohort study
Source: BMC Infect Dis. 2020 Mar 20;20:238. doi: 10.1186/s12879-020-04958-z (PMC7085166; doi:10.1186/s12879-020-04958-z)
Supplement: Supplementary file 2 — Additional file 2:Table S2. Stratified table for anemia risk and CD4 < 100 and HCV coinfection. [file 12879_2020_4958_MOESM2_ESM.docx]

Supplemental Table 2: Stratified table for anemia risk and CD4<100 and HCV coinfection

|  | Hazard ratio (95%CI) | |
| --- | --- | --- |
|  | HCV coinfection (n=2,179, n with CD4<100 =244) | No HCV coinfection (n=10,070, n with CD4<100 =842) |
| CD4<100 (ref CD4≥500) | 10.20 (8.01, 12.98) | 5.01 (3.39, 7.42) |
| Interaction between CD4<100 and HCV coinfection, p=0.001 | | |
